# Human-stimulated oocyte extract induces genetic and mitochondrial reprogramming of mesenchymal stromal cells

**DOI:** 10.1371/journal.pone.0232759

**Published:** 2020-05-26

**Authors:** Zaynab El-Gammal, Abdelrahman AlOkda, Sameh S. Ali, Asmaa Reda, Sameh Magdeldin, Ragaa Mansour, Nagwa El-Badri

**Affiliations:** 1 Center of Excellence for Stem Cells and Regenerative Medicine, Zewail City of Science and Technology, Cairo, Egypt; 2 Center for Aging and Associated Diseases, Helmy Institute of Medical Sciences, Zewail City of Science and Technology, Giza, Egypt; 3 Children's Cancer Hospital, Cairo, Egypt; 4 Molecular and Cellular Biology Department, Faculty of Sciences, Benha University, Benha, Egypt; 5 Proteomics and Metabolomics Research Program, Basic Research Department, Children’s Cancer Hospital, Kappelkinger, Egypt; 6 Egyptian IVF-ET center, Cario, Egypt; The University of Adelaide, AUSTRALIA

## Abstract

**Summary:**

Reprogramming autologous adult cells to pluripotent cells allows for relatively safe cell replacement therapy. This can be achieved by nuclear transfer, cell fusion, or induced pluripotent stem cell technology However, the epigenetic memory of the cell is considered as a great challenge facing the complete reprograming of cells by these methods. Introducing oocyte-specific factors into differentiated cells may present a promising approach by mimicking cellular reprogramming during fertilization.

**Methods:**

Human bone marrow mesenchymal stromal cells (hBM-MSCs) were cultured with different concentrations of human metaphase II (M II) oocyte extract (0.1, 1, 5, 10, 30 ng/μl). Reprogramming was assessed at various exposure times (1, 4, 7 days). Cells were tested for their proliferation rate, morphological changes, expression of pluripotency markers, expression of mesenchymal to epithelial transition markers, and mitochondrial rejuvenation. (mitochondrial localization, morphological changes, bioenergetics, transmembrane potential, and levels of reactive oxygen species, ROS).

**Results:**

Treatment of human BM-MSCs with 10 ng/μl oocyte extract resulted in increased cell proliferation, which was associated with the upregulation of the pluripotency genes *OCT-4*, *NANOG*, and *SOX-2* and a concomitant downregulation of mesenchymal-specific genes. MSCs exhibited small, immature round mitochondria with few swollen cristae localized proximal to the cell nucleus. This was accompanied by morphological cell changes, a metabolic shift towards oxidative phosphorylation, a high mitochondrial membrane potential, and increased ROS production.

**Conclusion:**

These data show that treatment with 10 ng/μl human MII-phase oocyte extract induced genetic and mitochondrial reprogramming of human BM-MSCs to a more embryonic phenotype.

## Introduction

Reprogramming autologous cells to pluripotent stem cells (PSCs) allows for relatively safe cell replacement therapy, disease modelling, and drug development studies. Pluripotency refers to the potential of specialized cells to give rise to different cell lineages. Reprogramming can be achieved by nuclear transfer, cell fusion or induced pluripotent stem cell (iPSC) technology (for example, by the overexpression of octamer-binding transcription factor 4 (OCT-4), Krueppel-like factor 4 (Klf4), sex-determining region Y- box 2 (SOX-2), and myelocytomatosis oncogene (c-Myc) (OKSM))[1–5].

However, inducing pluripotent stem cells from somatic cells using viral vectors to integrate OKSM genes into the host genome may increase the risk of tumor formation [6] Transient expression of the reprogramming factors using adenovirus vectors or plasmids, and direct delivery of reprogramming proteins were also mostly inefficient [7]. Additionally the epigenetic memory of the cell [8] and the already present repressive epigenetic marks might not allow transcription factors to bind properly [9].

Previous nuclear transfer experiments were effective in reprogramming somatic cells by transferring their nuclear contents into enucleated oocytes [6, 10–13]. Oocyte-specific factors in oocyte lysates provide the elements required for reprogramming [14]. The balance between metabolites and reactive oxygen species (ROS) in undifferentiated and differentiated stem cells provides intra- and inter-cellular environments that direct the epigenetic control of stem cell fate and pluripotency. This control was thought to occur through post-translational modifications of histones and DNA [15–17]. The dynamic balance among metabolic pathways, such as glycolysis and oxidative phosphorylation (OXPHOS), also influences self-renewal and lineage commitment in stem cells [18].

Earlier studies showed that Xenopus oocyte factors were used to direct the reprogramming of somatic cells into pluripotent cells [19–21]. Xenopus eggs were considered a model for mammalian oocytes, although their stable reprogramming was not usually achieved [19]. This was shown by the transient up-regulation of OCT-4 and guanylyl cyclase-activating protein (GCAP) expression; and the absence of SSEA-3, -4, Tra-1-60, and Tra-1-81 pluripotency cell surface markers [19].

In this work, we describe a novel method used to induce the genetic and mitochondrial reprogramming of somatic cells (bone marrow mesenchymal stromal cells, MSCs) treated with human oocyte extract. Reprogramming is determined based on the cellular proliferation rates, the expression of pluripotency markers and the expression of mesenchymal-to-epithelial transition (MET) markers. Furthermore, we aimed to assess the mitochondrial localization, morphological changes, bioenergetics, transmembrane potential, and levels of reactive oxygen species (ROS) in the reprogrammed cells.

## Results

### Oocyte extract preparation and protein quantification

Following the ultrasonic treatment of oocytes, the cell membrane was disrupted releasing the intracellular content of the oocyte (S1 Fig). Then, the protein content of the oocyte was measured by Qubit 3 fluorometer.

### Identification of hBM-MSCs

hBM-MSCs were characterized by their fibroblast-like morphology and adherence to the plastic surface. The cells uniformly expressed the surface markers CD105 and CD73 and lacked the expression of the CD45 surface marker (S2A, S2B and S3C Fig).

### Effect of oocyte extract treatment on hBM-MSCs proliferation

**Fig 1:**
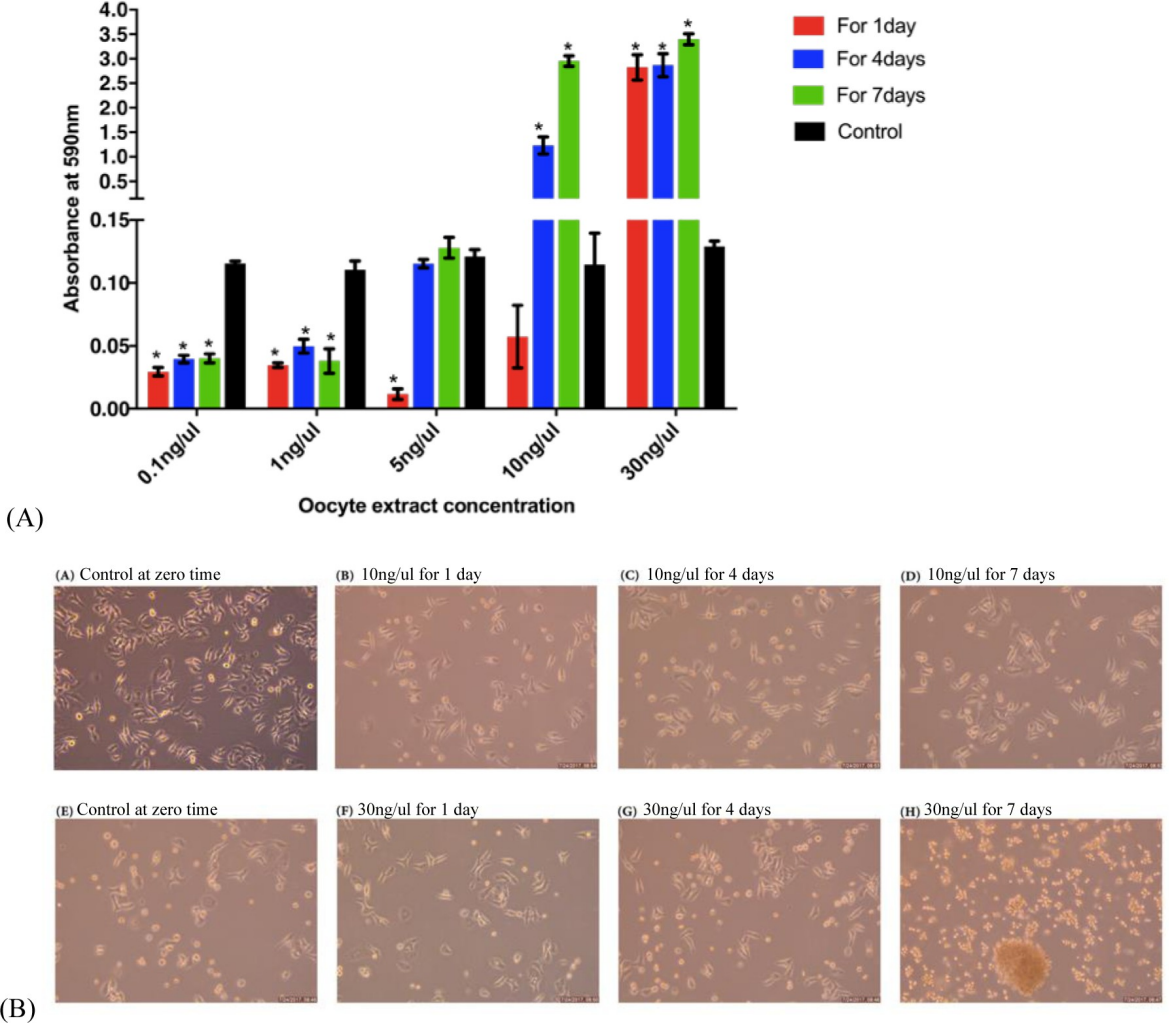
^**A**^ Proliferation rate of hBM-MSCs treated with 0.1, 1, 5, 10, and 30 ng/μl human oocyte extract compared with the untreated control cells. ^**B**^ Morphological features of hBM-MSCs before and after treatment with 10 and 30 ng/μl human oocyte extract for 1, 4 and 7 days.

Following the treatment with 0.1, 1, or 5 ng/μl oocyte extract, the proliferation of the hBM-MSCs decreased significantly. Compared to that of the control cells, the proliferation of the hBM-MSCs significantly increased following treatment with 10 or 30 ng/μl oocyte extract. Notably, proliferation increased in a time- and dose-dependent manner following treatment with 10 ng/μl oocyte extract (Fig 1).

### Effect of oocyte extract treatment on the morphology of hBM-MSCs

In assessing the concomitant morphological change following the treatment with either 10 or 30 ng/μl oocyte extract, the fibroblast-like shape of the hBM-MSCs treated with 10 ng/μl oocyte extract for 1 day, 4 days, and 7 days remained unchanged compared to that of the control (Fig 1B). The hBM-MSCs treated with 30 ng/μl oocyte extract showed a change from a fibroblast-like shape to a spherical shape with longer treatment times. Additionally, notably, the 7-day treatment yielded spherical cell colonies resembling ESCs (Fig 1B).

### Effect of oocyte extract treatment on the expression of pluripotency genes in hBM-MSCs

**Fig 2:**
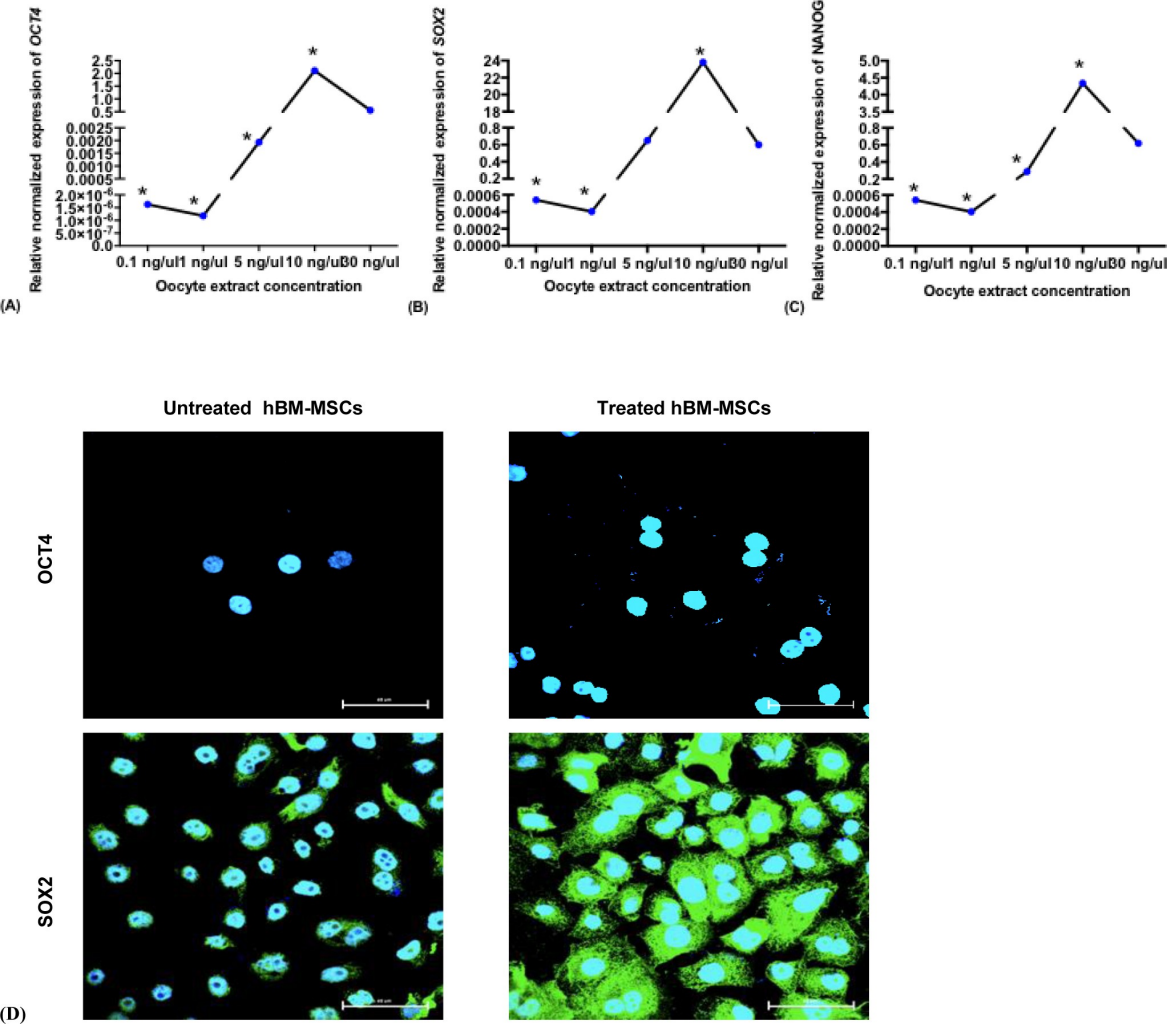
Relative expression of pluripotency markers. ^**A**^ *OCT4*, ^**B**^ *SOX2*, ^**C**^ *NANOG* in hBM-MSCs treated with 0.1, 1, 5, 10, and 30 ng/μl human oocyte extract for 4 days compared to their untreated control. ^**D**^ Confocal microscopy of hBM-MSCs treated with 10 ng/μl human oocyte extract for 4 days. A single optical section of confocal z-stack series merged by ^**Upper panel**^ OCT-4 (Alexa Fluor 488) and Hoechst immunostain and ^**Lower panel**^ SOX-2 and Hoechst immunostain. Original magnification x63.

Following the treatment of hBM-MSCs with 0.1, 1, 5, 10 or 30 ng/μl oocyte extract, only the 10 ng/μl concentration resulted in a significant upregulation in the expression of the pluripotency genes OCT-4, SOX-2, and NANOG compared to the level in the untreated controls (Fig 2).

Based on these data, we selected the 10 ng/μl concentration for further assessment of pluripotency gene expression over time. Due to the interesting finding that treating hBM-MSCs with 30 ng/μl for 7 days yielded spherical cell colonies resembling ESCs, we also investigated pluripotency gene expression over the time course.

### Effect of oocyte extract treatment on the expression of pluripotency genes in hBM-MSCs over the time course

**Fig 3:**
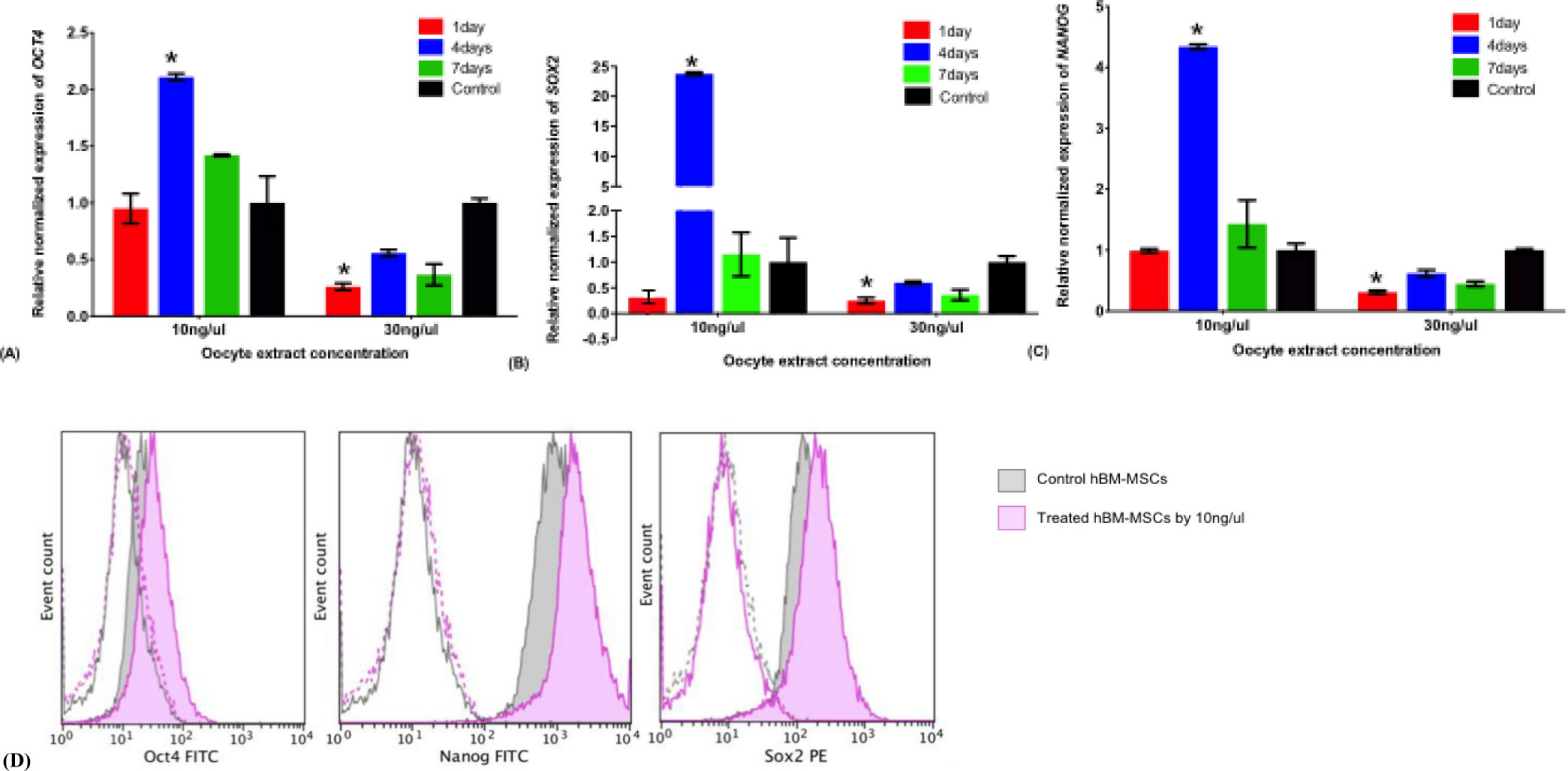
Expression of pluripotency markers. ^**A, B, C**^ Relative expression of pluripotency markers *OCT4* ^**A**^, *SOX2* ^**B**^ and *NANOG* ^**C**^ in hBM-MSCs treated with 10 and 30 ng/μl human oocyte extract for 1, 4 and 7 days assessed by real time PCR. ^**D**^ Flow Cytometry analysis of pluripotency markers OCT-4, NANOG, and SOX2 in hBM-MSCs oocyte extract-treated group for 4 days compared with the untreated group.

Following the treatment of the hBM-MSCs with 10 ng/μl oocyte extract, the expression of the pluripotency genes *OCT-4*, *SOX-2*, and *NANOG* did not significantly change after 1 and 7 days of oocyte extract treatment but significantly increased after 4 days of treatment compared to that in the control cells (2, 23.8, and 4.4-fold, respectively) (Fig 3A, 3B and 3C). These data were confirmed by the flow cytometry and confocal microscopy analysis (Figs 3D, 3E, 3F and 2D). However, the treatment of the hBM-MSCs with 30 ng/μl oocyte extract showed no significant upregulation of the pluripotency markers compared to their levels in the control at the three time points (Fig 3A, 3B and 3C).

### Effect of oocyte extract treatment on mesenchymal-to-epithelial transition (MET) marker expression

**Fig 4:**
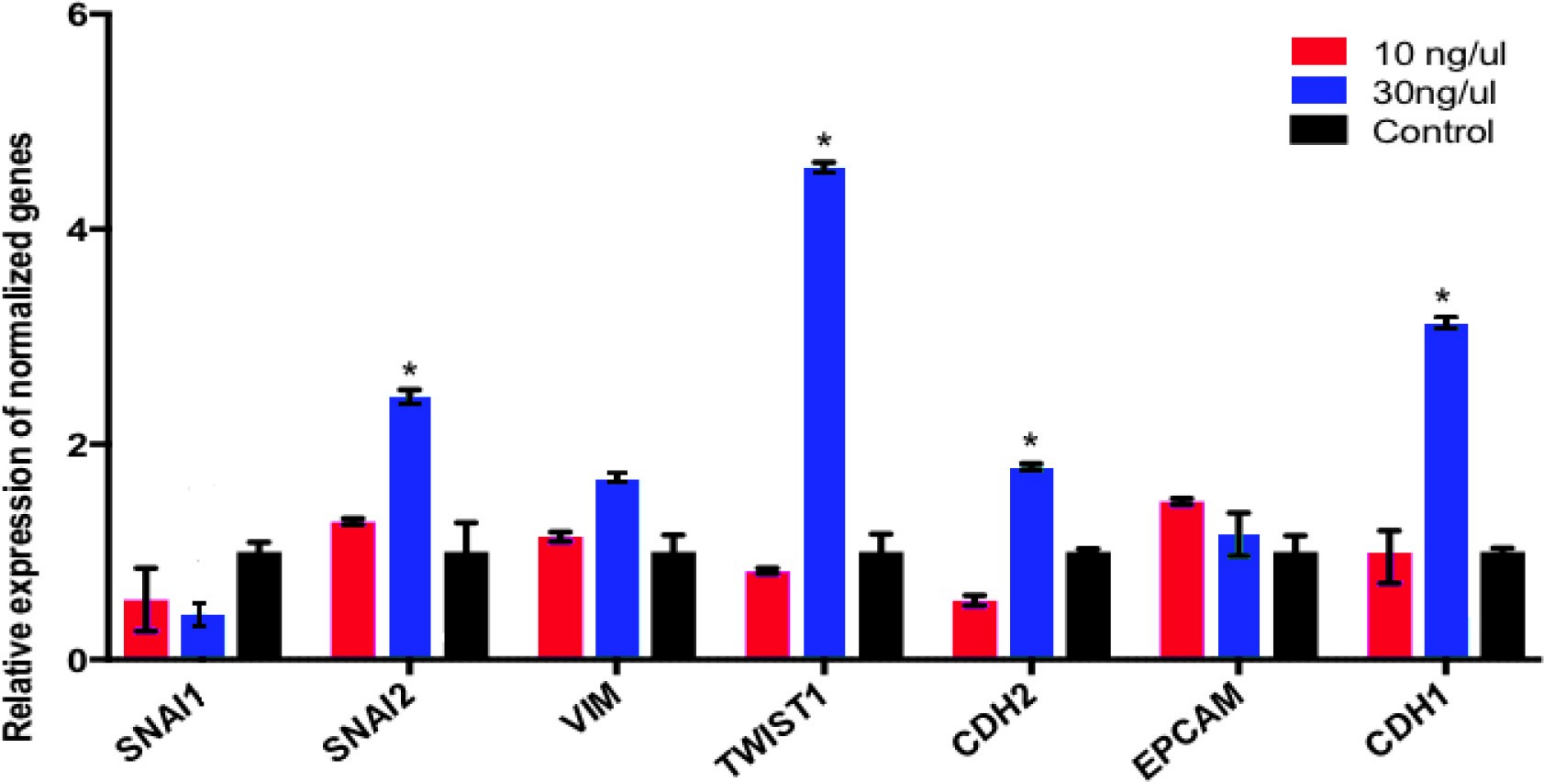
Relative expression of mesenchymal-to-epithelial transition in hBM-MSCs treated with 10 and 30 ng/μl human oocyte extract for 4 days. Relative expression of *SNAI1*, *SNAI2*, *VIM*, *TWIST1*, *CDH2*, *EPCAM* and *CDH1*.

Following the treatment of the hBM-MSCs with 10 ng/μl oocyte extract, some mesenchymal markers were downregulated (SNAI1 and CDH2 encoding Snail and N-cadherin proteins), while other markers remained unchanged (SNAI2, TWIST1, and VIM encoding Slug, Twist, and Vimentin proteins). Regarding the epithelial markers, EPCAM (encoding Epcam protein) was upregulated, while CDH1 (encoding E-cadherin proteins) remained unchanged. Following the treatment of the hBM-MSCs with 30 ng/μl oocyte extract, SNAI2, VIM, TWIST1, CDH2, and CDH1 were significantly upregulated, but SNAI1 and EPCAM remained unchanged (Fig 4).

### Assessment of reprogramming factor- independence

**Fig 5:**
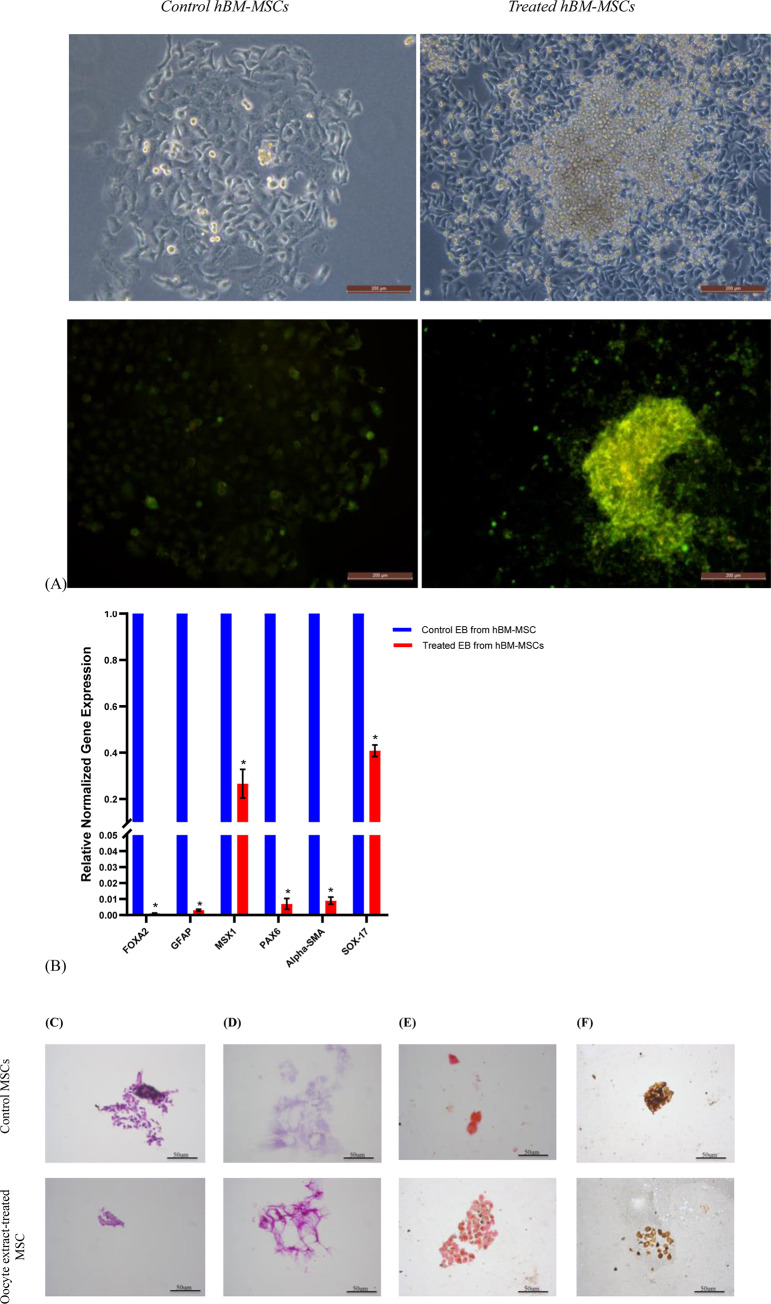
Pluripotency of treated and control hBM-MSCs. ^**A** Upper pannel^ Embryoid body (EB) formation by control and oocyte extract treated hBM-MSCs, ^A Lower pannel^ EB stained with alkaline phosphatase. ^**B**^ Relative expression of three germ layer differentiation markers in hBM-MSCs treated with 10 ng/μl human oocyte extract for 4 days. ^**C, D, E, F**^ Histological analysis of hBM-MSCs control and treated by 10ng/ul oocyte extract for 4 days. hBM-MSCs were stained by ^**C**^ H&E; ^**D**^ PAS to assess differentiation into hepatocytes; ^**E**^ Safranin O to assess the chondrogenic differentiation; and ^**F**^ Silver impregnation-LFB PAS stain to assess the neural differentiation.

Following the treatment of hBM-MSCs with 10 ng/μl oocyte extract for 4 days, hBM-MSCs displayed enhanced embryoid body formation (Fig 5A), as confirmed by alkaline phosphatase staining (Fig 5B). Reprograming efficiency was 0.0025% as assessed by the number of AP^+^ colonies per number of plated cells.

Fig 5B, shows significant downregulation of the three germ layers, endodermal, mesodermal, and ectodermal markers in hBM-MSCs treated by 10 ng/ μl oocyte extract for 4 days. Upon treatment by 10ng/ul oocyte extract, hBM-MSCs exhibited a higher ability to differentiate to chondrocytes (Fig 5E), and neural cells (Fig 5F), while control untreated cells failed to differentiate into neural cells (Fig 5F). However, neither control nor treated cells were differentiated to hepatocytes using specific differentiation medium (Fig 5D).

Based on the promising results observed following the treatment of the hBM-MSCs with 10 ng/μl oocyte extract for 4 days, we selected this group for further analysis to assess mitochondrial function and the metabolic switch.

### Effect of oocyte extract treatment on mitochondrial bioenergetics

**Fig 6:**
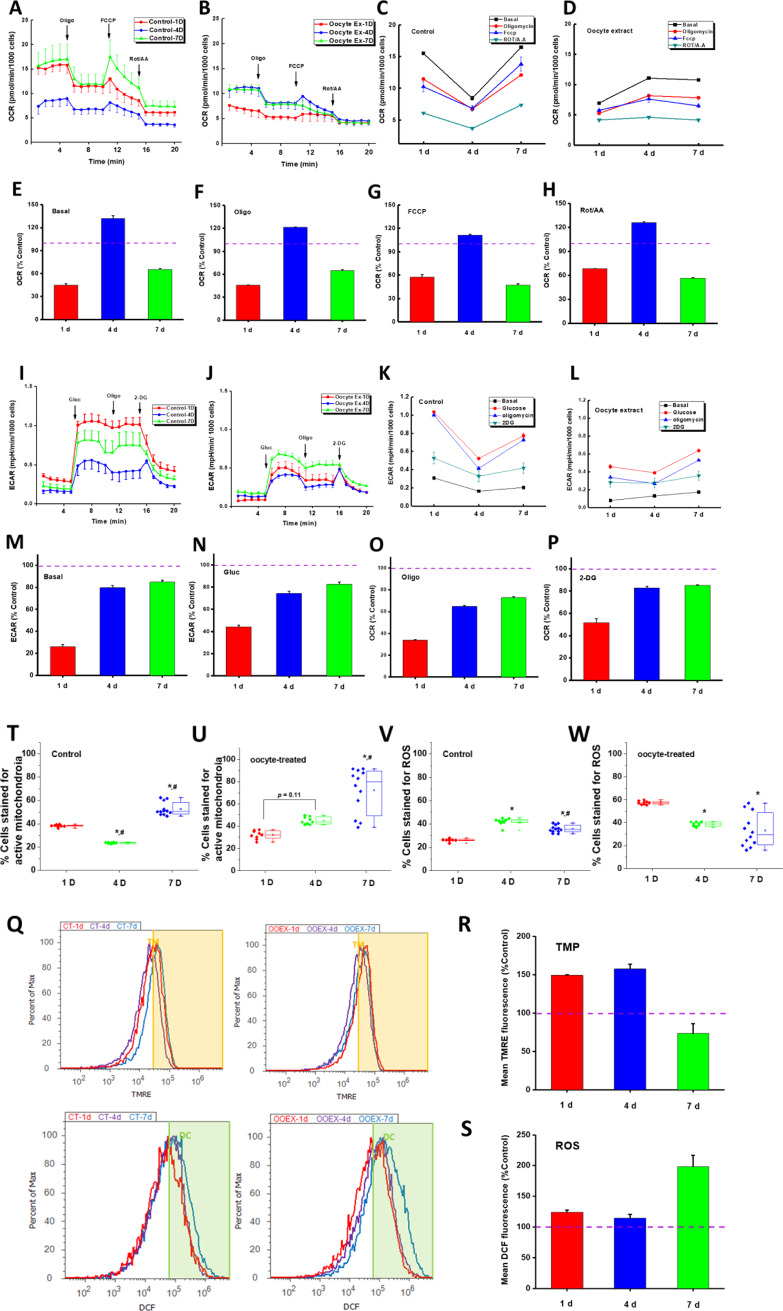
^A-H^ Mitochondrial metabolic profiling and changes induced by oocyte extract treatment of MSCs for 1, 4, and 7 days. Monolayers of viable MSCs in DMEM buffer were subjected to Seahorse analyses in control cells ^A,C^ and Oocyte-treated cells ^B,D^. To quantify changes in glucose utilization upon treatment, we calculated ratios of oxygen consumption rates (OCR) which reflect oxidative phosphorylation capacities at various conditions: Basal ^E^, ATP-synthase inhibited by oligomycin ^F^, uncoupled state by FCCP ^G^, and complex I & III inhibited state ^H^. Additions were 1 μM oligomycin at *Oligo*, 0.5 mM carbonyl cyanide-4-(trifluoromethoxy) phenylhydrazone at *FCCP*, and 0.5 μM rotenone + 1 μM Antimycin A at *Rot/AA*; see Methods‘ Section for detailed description. ^I-P^ Glycolytic profiling and changes induced by oocyte extract treatment of MSCs for 1, 4, and 7 days as described above for control cells ^I,K^ and Oocyte-treated cells ^J,L^. To monitor glucose utilization during glycolytic metabolism, we determined extracellular acidification rate (ECAR) during various conditions: Basal lactate formation by glucose-starved MSC ^M^, lactate formation by cells metabolizing unlimited glucose supply ^N^, maximum glucose utilization due to mitochondrial inhibition by oligomycin ^O^, and upon inhibiting glycolysis by 2-deoxy-d-glucose (*2-DG*) ^P^. Additions were 5 mM glucose at *gluc*, 1 μM oligomycin at *Oligo*, and 50 mM 2-DG; see Methods‘ Section for detailed description. ^Q-W^ Flow cytometry analyses of temporal changes in mitochondrial transmembrane potential (TMP) and levels of ROS induced by oocyte extract treatment of MSCs after 1, 4, and 7 days. In the bar graphs, mitochondrial TMP in treated cells was evaluated at all time points by comparing the mean TMRE fluorescence intensity as percentage of control cells and collected over three independent runs each with three replicates ^R^. ROS levels were similarly calculated as percentage of mean DCF fluorescence intensities in control cells ^S^. We also analyzed changes in the fractions of cell populations that were positively stained with TMRE and DCF and reflecting greater contents of active mitochondria and ROS ^T-W^. * denotes statistical significance (ANOVA, p < 0.05) relative to the 1-d time point and # denotes that relative to the 4-d time point.

Using the Seahorse XF-24 Metabolic Flux Analyzer, we determined the mitochondria-mediated oxygen consumption rates (OCR, Fig 6A–6H) and extracellular acidification rates (ECAR, Fig 6I–6P) caused by lactate release after 1, 4, and 7 days of oocyte extract treatment.

### Oxygen consumption rate

In the control hBM-MSCs, an overall moderate increase in the oxidative phosphorylation parameters was observed after 4 days (Fig 6A). However, after 7 days, mitochondrial NADH-linked respiration was strongly suppressed. The treatment with oocyte extract caused a universal metabolic suppression, but this suppression was more effective after 1 and 7 days of treatment (Fig 6B).

After normalizing the various respiratory parameters recorded in the treated cells to parallel values obtained in the control cells, a strong general suppression of mitochondrial respiration was observed after 1 and 7 days of treatment (Fig 6E–6H). However, the oocyte treatment seems to transiently enhance mitochondrial respiration after approximately 4 days of treatment. All changes were statistically significant compared to control levels using a one-way ANOVA (N = 3 replicates and 5 repetitions, p<0.05). Interestingly, the observed time profiles of the mitochondrial metabolic changes approximately corresponded to the trends observed in the parallel changes in the pluripotency gene markers *OCT-4*, *NANOG*, and *SOX-2* (Fig 3).

Nevertheless, the glycolytic activities were significantly downregulated following the treatment with 10 ng/μl oocyte extract (Fig 6M–6P). This effect was more pronounced after 1 day of treatment with approximately 70% suppression of the basal and maximal glycolytic activities.

### Effect of oocyte extract treatment on transmembrane potential and reactive oxygen species levels

To confirm the results of the mitochondria-mediated metabolic changes by the oocyte extract treatment and explore the concomitant changes in the levels of reactive oxygen species (ROS), we analysed the mitochondrial transmembrane potential and ROS levels in MSCs under various conditions using flow cytometry (Fig 6Q).

The oocyte treatment increased the TMRE fluorescence relative to that in the control cells, especially after 1 and 4 days, and a >50% increase was observed in the mean fluorescence values (Fig 6R). Furthermore, the oocyte extract caused a parallel general increase in the ROS levels at all time points, especially after 7 days (Fig 6S). These results suggest that the oocyte extract treatment of the MSCs for 7 days generated hypo-polarized mitochondria that leak greater levels of ROS relative to untreated cells (Fig 6R and 6S). However, there has been a significant increase in the number of cells possessing active mitochondria (Fig 6U). This may be taken to imply that oocyte treatment has changed the metabolic phenotype of the MSCs to elicit more viable cells with greater numbers of less metabolically active mitochondria relative to untreated MSCs. Interestingly, these changes observed after 7 days of treatment led to significant increase in ROS levels (6S Fig), which are consistently reported as necessary factors enhancing cell proliferation [22]. One may suggest that the observed changes in stemness genes under oocyte treatment may be responsible for the observed metabolic remodeling [23]. In a relevant recent study, it has been shown that aggregation of hMSC changes mitochondrial morphology and reduces mitochondrial membrane potential [24]. Alternatively, the overall metabolic suppression following the oocyte treatment may reflect a bioenergetic shortage that hampers the mesenchymal-to-epithelial transition despite the enhanced expression of genes that mark pluripotency.

### Effect of oocyte extract treatment on mitochondrial morphology and localization

**Fig 7:**
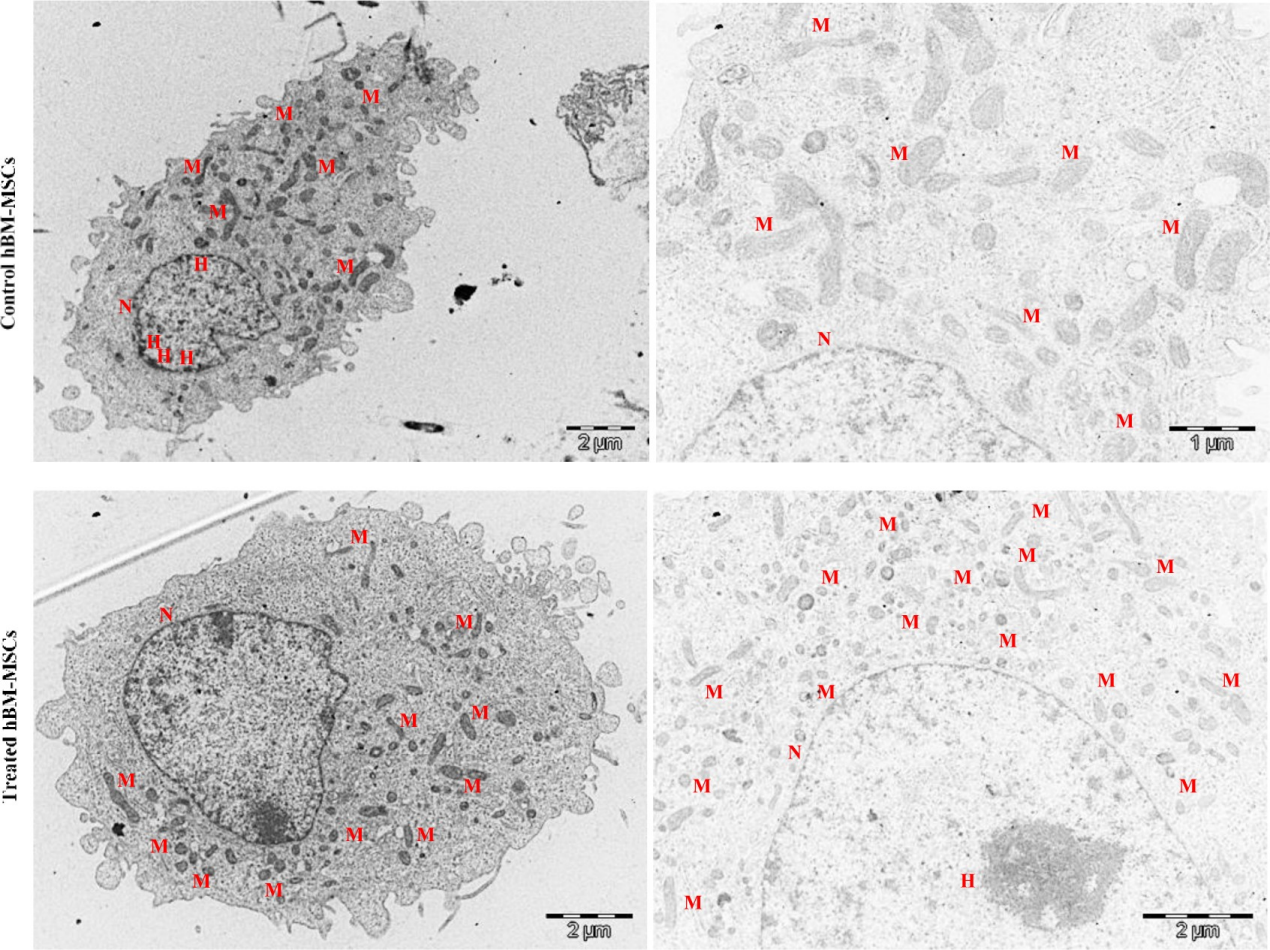
Transmission electron microscopy (TEM) of hBM-MSCs treated with 10 ng/μl human oocyte extract for 4 days showing mitochondrial distribution and morphology. N: Nucleus, M: Mitochondria.

Transmission electron microscopy (TEM) analysis of the hBM-MSCs control revealed elongated cells (14 μm length and 6.9 μm diameter), small and spread heterochromatin foci, and elongated mitochondria that were spread in the cytoplasm (Fig 7). In contrast, hBM-MSCs treated with 10 ng/μl oocyte extract revealed more rounded cells (12.9 μm length and 10.1 μm diameter) and large heterochromatin foci localized near the nuclear membrane. The treated cells also showed small and round mitochondria, with low numbers of swollen cristae localized proximal to the cell nucleus (Fig 7).

### Identification of oocyte extract proteins

**Fig 8:**
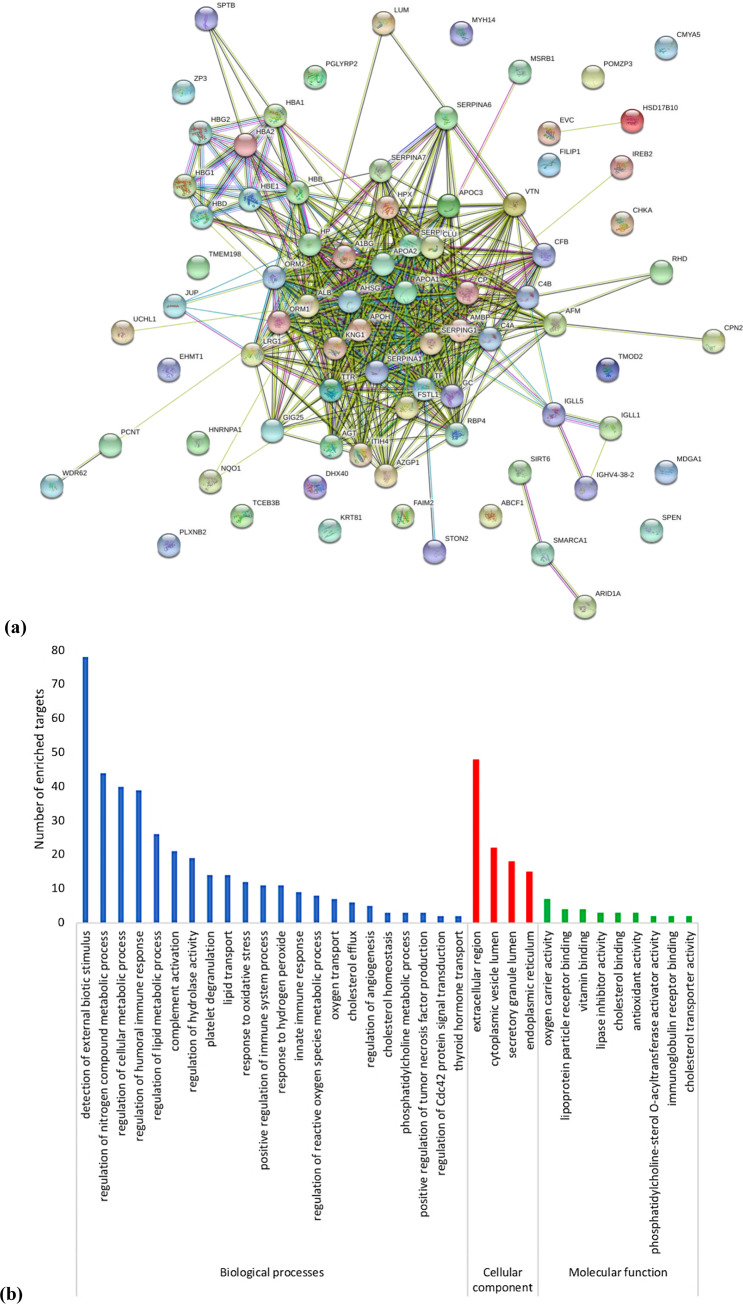
The functional enrichment analysis of the protein content of the oocyte extract. (a) STRING network. (b) DAVID enrichment analysis represents the involved biological processes (GO), cellular components, and molecular functions.

It was previously reported that factors involved in cell reprogramming can be identified by means of the protein activity [20, 25].The annotation of gene function of oocyte extract proteins was performed with GO analysis using STRING database. The results suggested that oocyte extract proteins participated in oxygen carrying, lipoprotein binding, cholesterol binding, antioxidant activity, and immunoglobulin receptor binding (Fig 8B). Also, GO analysis showed that oocyte extract proteins are located in different areas: extracellular, cytoplasmic vesicle lumen, secretory granule lumen, and endoplasmic reticulum.

To illustrate the interaction between oocyte extract proteins and the possible involved pathways in this reprograming effect, we constructed the interaction network containing nodes corresponding to oocyte proteins using STRING database (Fig 8A). Oocyte extract proteins were involved in the regulation of metabolic process, humoral immune response, complement activation, hydrolase activity, lipid transport, response to oxidative stress, and cholesterol efflux.

## Discussion

Reprogramming differentiated cells into more embryonic cells, capable of multilineage differentiation potential is an important goal for regenerative medicine as it provides opportunities for relatively safe cell replacement therapy. Xenopus eggs were used to reprogram human differentiated cells (human 293T kidney cells, and human primary leucocyte cells**)** as an alternative for mammalian oocytes that are difficult to obtain in large quantities [19]. However, this reprogramming was only transient. Thus, in this study, we aim to assess whether human stimulated oocyte extract could induce reprogramming of hBM-MSCs into more pluripotent cells, as determined by morphological, genetic and metabolic changes.

Cellular reprogramming consists of the following three phases: initiation, maturation and stabilization [26]. Expression changes in response to reprogramming factors begin immediately; however, the pluripotent state is not achieved until several days later [26–28]. Our data confirm that the treatment of hBM-MSCs with 10 ng/μl oocyte extract for 4 days stimulates the cells to enter the three phases of reprogramming. The initiation phase of reprogramming was achieved by the activation of cell proliferation, and the change in cell shape that can by itself trigger epigenetic modifications regulating reprogramming [27, 29]. This change in morphology has been shown to be an important early event that occurs in cells that reach the pluripotent state [41].

Treatment with 10 ng/μl oocyte extract for 4 days transitioned hBM-MSCs into the maturation phase of reprogramming, which is characterized by the onset of the first pluripotency-associated genes, such as endogenous *OCT-4*, followed by *NANOG* [26, 30, 31]. Overexpression of *OCT-4*, *SOX-2* and *NANOG* is known to maintain the pluripotent state of stem cells [32–34] and prevent hESC differentiation [35]. For instance, OCT-4 controls both extra-embryonic and epiblast-derived cell fates, NANOG represses embryonic ectoderm differentiation, and SOX-2 and SOX-3 repress mesoderm differentiation [36]. This increase in the cellular proliferation and pluripotency upon treatment is at least in part due to Apolipoprotein A-I, Apolipoprotein A-II, Apolipoprotein C-III, Clusterin, present in the oocyte extract, that are involved in the lipid biosynthesis [37, 38]. Moreover, cholesterol metabolism plays a key role in the cellular reprograming [23, 39].

According to several studies, the acquisition of maturation genes alone does not guarantee the complete reprogramming of cells [30, 40, 41]. The upregulation of the *SOX-2* marker (considered a stabilization-phase gene) and the independence from the reprogramming factors demonstrate that cells are able to successfully transition to the stabilization phase and acquire the full pluripotency signature independently of extrinsic reprogramming factors [8, 30, 40–46]. This reprograming effect may be due to complement 4A, complement 4B, complement factor B, clusterin, carboxypeptidase N Subunit 2, immunoglobulin Lambda Like Polypeptide 5, c1 inhibitor, vitronectin, present in the oocyte extract, that are responsible for the activation of complement and coagulation cascade. This is a major component of the innate immunity system whose activation is necessary for efficient nuclear reprograming [47].

Because mitochondria are responsible for energy production through OXPHOS and cell signalling through ROS production, opposite remodelling of the mitochondrial network (mitochondrial rejuvenation) is indispensable for efficient reprogramming into induced PSCs (iPSCs) [48–51]. These metabolic pathways can lead to stem cell reprogramming by affecting the epigenetics, proliferation, and differentiation pathways [52]. Furthermore, mitochondria can exhibit multiple different morphologies and subcellular localizations depending on their activity. Actively respiring mitochondria exist as a filamentous network with elongated shapes that are densely packed with cristae to allow for a greater surface area to house electron-transport chain complexes [53]. Inactive mitochondria are small and round with a few swollen cristae [49, 54–56]. This is in accordance with our data that showed that upon treatment of hBM-MSCs by oocyte extract, mitochondria became more rounded. Then, we investigated the effect of human oocyte extract on mitochondrial rejuvenation by assessing the mitochondrial bioenergetics, transmembrane potential, ROS production, shape, and location. The high mitochondrial membrane potential, especially after 1 and 4 days treatment with oocyte extract, is crucial for maintaining pluripotency and self-renewal. Also, pluripotent cells with high membrane potential can differentiate into the three germ layers in contrast to those with low membrane potential that can differentiate only into mesoderm [36, 57–59]. Moreover, high mitochondrial membrane potential is important for the regulation of the redox potential to be ideal for lipids and amino acids biosynthesis.

This is also accompanied by an increase in the OxPhos. This increase is crucial in the early phases of reprograming to generate ROS that, in turn, induce a cascade of transcription factors to initiate the metabolic shift [60]. Also, the increase in OXPHOS along with the increase in Nanog expression may signal the involvement of Estrogen-related receptor beta (ESRRB) in this reprogramming [61]. ESRRB is indispensable for the activation of NANOG expression and the maintenance of pluripotency since it is the nuclear receptor that allows the binding of nuclear receptor co-activator (NCOA3) in human oocytes to the NANOG promoter [62–66]. However, in later stages of nuclear reprogramming, OxPhos increase must be followed by an up-regulation of antioxidant enzymes that reduce ROS [67]. This is why the activation of PPAR signaling pathway is crucial for the reprograming of metabolic memory. We found that oocyte extract is enriched in proteins involved in peroxisome proliferator-activated receptor (PPAR) signaling pathway. PPARγ protects the cell from the effect of elevated ROS level by recruiting the PPARγ coactivator 1-alpha (PGC-1α), the chief regulator of ROS scavenging enzymes [68]. Further assessment of the activation of PPAR signaling in the treated cells is required.

Noteworthy, both glycolytic and OxPhos can support pluripotent stem cell growth [69]. The dependence on OxPhos in pluripotent stem cells is based on the composition of the medium in which these cells are cultured. Lipid supplementation is proved to increase the oxidative phosphorylation [69].

The observed metabolic reprograming of treated cells may be at least in part due to hydrolases, in the oocyte extract, that are involved in the induction of autophagy and the subsequent metabolic reprograming [70].

This reprogramming effect was not duration- or dose-dependent in our experience.

## Conclusion

We showed that treatment with 10 ng/μl human- phase II oocyte extract induces genetic and mitochondrial reprogramming of hBM-MSCs. This reprogramming was achieved through the three phases of reprograming as indicated by the induction of cell proliferation; expression of embryonic stem cell markers, such as *OCT-4*, *SOX-2*, and NANOG; the inhibition of somatic program, the change in cell shape, and the independence from the reprogramming factors. This reprogramming however is partial, as shown by the presence of heterochromatic foci, the limited developmental potential, the increase of OxPhos, membrane potential, and ROS production.

Further assessing these reprogrammed cells to identify additional markers, performing epigenetic and transcriptome profile analyses, and testing their differentiation abilities could provide better insight into the reprogramming function of the oocyte microenvironment.

## Material and methods

### Ethical approvals

The Egyptian *In Vitro* Fertilization Center Internal Review Board (IRB) approved the study protocol. Informed consent was obtained from all individual participants in the study.

### Oocyte extract preparation

The oocyte extract was prepared from discarded oocytes following failed *in vitro* fertilization. Oocyte extract was performed using an ultrasonic probe type sonicator (Q700 Sonica, USA). The protein content in the pooled oocyte extract was quantified using a Qubit 3.0 fluorometer and a Qubit protein assay kit (Life technologies, USA).

### Culture of hBM-MSCs

hBM-MSCs were a gift from the tissue culture lab of the Egyptian company for production of vaccines, sera, and drugs (Vacsera, Giza, Egypt). MSCs were cultured in complete culture medium (45 ml DMEM low glucose without L-glutamine (Lonza, Switzerland), supplemented with 5 ml fetal bovine serum FBS (Biowest, France), 0.5 ml antibiotic antimycotic (Lonza, Switzerland), and 0.5 ml L-glutamine (Lonza, Switzerland)) under standard culture conditions (37°C and 5% CO2). Upon reaching 70–80% confluency, the cells were passaged with trypsin-EDTA (0.25%) (Lonza, Switzerland) and re-suspended in complete culture medium.

### hBM-MSC culture with oocyte extract

Purified 0.5×10^6^ hBM-MSCs cultured in 25 cm^2^ culture flasks were washed twice with 2 ml PBS without Ca^+2^ or Mg^+2^ (Lonza, Switzerland), and oocyte extracts at concentrations of 0.1, 1, 5, 10, or 30 ng/μl were added accordingly. The cells were incubated for 1 day, 4 days, or 7 days. Pluripotency markers were assessed as described below.

### Cytotoxicity assay

Cell viability was assessed using a modified 3-(4,5-dimethylthiazol-2-yl)-2,5diphenyl-tetrazolium bromide (MTT) assay. Briefly, 10^5^ hBM-MSCs were plated in 6-well plates with 4 ml complete culture medium per well. After 24 hours, the oocyte extract was applied to wells containing cells, except for the control group. The assessment was performed 1 day, 4 days, and 7 days after the treatment with the oocyte extract.

To avoid any interference with the colorimetric MTT assay, the oocyte extract was added to wells containing complete culture medium alone as a blank. On the following day, 320 μl of 5 mg/ml MTT (Serva, Germany) were added to each well, and the plates were incubated for 3.5 hours. Finally, the medium was carefully removed, and 2400 μl of solubilization solution (dimethyl sulfoxide (Serva, Germany)) were added, followed by 15 min shaking using an orbital shaker (Thermofisher Scientific, USA). The absorption readings were performed at 490 nm using ELISA plate reader (Biotek, USA).

### RT-PCR

RNA extraction was achieved using an RNeasy Mini Kit total RNA extraction kit (Qiagen, Germany). In total, 10 μl total RNA, 1 μl antisense primer (20 pmol), and 1 μl reverse transcriptase enzyme were used for the preparation of the cDNA (15 min at 42°C). In total, 5 μl cDNA, 2X SYBR Green PCR Master Mix (Applied Biosystems, USA) and 5 pmol of each primer (Table 1) were used to perform the quantitative RT-PCR analysis. The amplification conditions included an initial denaturation step (15 min at 95°C), followed by 40 cycles of denaturation (15 sec at 94°C), annealing (60°C for 30 sec) and extension (30 sec at 72°C).

**Table 1:** Primer sequence of pluripotency markers genes used in real time PCR.

Gene	Forward primer	Reverse primer
*OCT4*	TGTACTCCTCGGTCCCTTTC	TCCAGGTTTTCTTTCCCTAGC
*SOX2*	GCTAGTCTCCAAGCGACGAA	GCAAGAAGCCTCTCCTTGAA
*NANOG*	CAGTCTGGACACTGGCTGAA	CTCGCTGATTAGGCTCCAAC
*CDH2*	GGTGGAGGAGAAGAAGACCAG	GGCATCAGGCTCCACAGT
*SNAI1*	ACCACTATGCCGCGCTCTT	GGTCGTAGGGCTGCTGGAA
*SNAI2*	TGTTGCAAGTGAGGGCAAGAA	GACCCTGGTTGCTTCAAGGA
*TWIST1*	CGGGAGTCCGCAGTCTTA	GCTTGAGGGTCTGAATCTTG
*VIM*	TGTCCAAATCGATGTGGATGTTTC	TTGTACCATTCTTCTGCCTCCTG
*CDH1*	GTCACTGACACCAACGATAATCCT	TTTCAGTGTGGTGATTACGACG
*EPCAM*	CGCAGCTCAGGAAGAATGTG	TGAAGTACACTGGCATTGACG
*ACTB*	AGAGCTACGAGCTGCCTGAC	AGCACTGTGTTGGCGTACAG
*PAX-6*	TGGTATTCTCTCCCCCTCCT	TAAGGATGTTGAACGGGCAG
*GFAP*	AGAAGCTCCAGGATGAAACC	AGCGACTCAATCTTCCTCTC
*Alpha SMA*	CCGACCGAATGCAGAAGGA	ACAGAGTATTTGCGCTCCGAA
*MSX1*	CCTCTTTGCTCCCTGAGTTCA	GGGACTCTTCCAGCCACTTTTT
*FOXA2*	CCGTTCTCCATCAACAACCT	GGGGTAGTGCATCACCTGTT
*SOX-17*	CGCTTTCATGGTGTGGGCTAAGGACG	TAGTTGGGGTGGTCCTGCATGTGCTG

Using the comparative Ct method (Gen5 2.05 software), real-time data were used to calculate the relative expression of the pluripotency markers and MET markers. β-actin was used as a housekeeping gene to enable the normalization of all values. The values are reported as the fold change over the background levels.

### Flow cytometry

#### Characterization of hBM-MSCs

hBM-MSCs from passage 14 were trypsinized using 0.25% trypsin/EDTA (Life Technologies, USA). To prevent non-specific binding, the cells were re-suspended for 10 min in blocking solution and PBS supplemented with 1% FBS (Life Technologies, USA) before centrifugation at 200 xg for 5 min. The cells were re-suspended in blocking solution and stained with PerCP anti-CD105, FITC anti-CD73 and APC anti-CD45 monoclonal antibodies (Becton Dickinson, USA) for 30 min at 4°C in the dark. To remove the unbound antibodies, the cells were centrifuged at 2000 rpm for 10 min.

#### Expression of pluripotency genes

Untreated hBM-MSCs and oocyte extract-treated hBM-MSCs were trypsinized and briefly centrifuged. The cells were fixed in 4% paraformaldehyde in Dulbecco PBS (DPBS) (Lonza, Switzerland) for 30 min and permeabilized with 0.1% Triton X-100 (Acros Organics, USA) in DPBS for 30 min after washing with DPBS three times. The cells were incubated in blocking solution and 4% bovine serum albumin (BSA) (Serva, Germany) for 30 min and stained with the primary antibodies (BD Pharmingen^™^ Alexa Fluor® 488 Mouse anti-Human OCT3/4, BD Pharmingen^™^ Alexa Fluor® 488 Mouse anti-Human SOX-2, BD Pharmingen^™^ Alexa Fluor® 555 Rabbit anti-Human NANOG) (Becton Dickinson, USA) for 1 hour. After another thorough washing with DPBS, the cells were labelled with the appropriate secondary antibody (BD Pharmingen^™^ FITC Goat Anti-Rabbit IgG or BD Pharmingen^™^ FITC Rat Anti-Mouse IgE) (Becton Dickinson, USA) for 2 hours. Finally, the cells were analysed using a FACSCalibur (Becton Dickinson, USA) following standard procedures using Cell Quest Pro Software (Becton Dickinson, USA).

### Confocal fluorescence microscopy immuno-staining

After treatment with oocyte extract, hBM-MSCs were seeded onto glass slides pre-coated with poly-D-lysine (Sigma-Aldrich, USA). The cells were fixed in 4% paraformaldehyde, permeabilized with 0.1% Triton X-100, and blocked with 1% BSA. The cells were stained with monoclonal antibodies specific to OCT-4, SOX-2, and NANOG antigens. The cells were labelled with the appropriate Alexa Fluor^®^ secondary antibodies (Molecular Probes, USA) and counterstained with Hoechst 33342 (Molecular Probes, USA) to visualize the cell nucleus. The cells were imaged under a 63X objective with a Leica DMi8 inverted laser scanning confocal microscope (Leica microsystems, Germany).

### Measurement of intracellular reactive oxygen species and mitochondrial membrane potential

Flow cytometric analysis of cells stained with 2’,7’-dichlorodihydrofluorescein diacetate (DCF-DA, Sigma) tetramethylrhodamine ethyl ester (TMRE, Sigma) was performed using an Attune^®^ acoustic focusing cytometer system (Applied Biosystems; now Thermo Fischer Scientific) and Attune software. In each analysis, 20000 gated events were observed. The hBM-MSCs were treated with oocyte extract at a concentration of 10 ng/μl for 1, 4, and 7 days and compared with control untreated cells. The quantification of intracellular ROS was performed using DCF, and the measurement of the mitochondrial transmembrane potential (ΔΨm) was performed using TMRE dye. The cells were co-stained with the two dyes (1 μM DCF and 500 nM TMRE). The quantification of the mean fluorescence or cell populations were performed in 3 separate experiments and statistically compared using ANOVA.

### Seahorse analysis

The two main key metabolic parameters, i.e., mitochondrial OXPHOS and cellular glycolysis, were assessed using a Seahorse XF-24 Flux Analyzer (Seahorse Biosciences) according to the manufacturer’s instructions. Prior to each assay, the XF sensor cartridges were hydrated. Oocyte-treated and untreated BM-MSCs were seeded and left to grow to obtain a monolayer of cultured cells (4×10^4^ cells in 100 μl media per well). The cells were allowed to attach for 4 hours, 150 μl MSC media were added, and four wells of the cartridge were untreated (as a blank control) for the background corrections. In total, 1 ml of Seahorse Bioscience XF-24 calibrant pH 7.4 was added to each well and placed below the cartridge. The cells were allowed to grow overnight at 37°C under 5% CO_2_. The cells within every well were checked 16 hours after the initial seeding under a microscope to verify the uniform distribution and confluency. The growth medium was aspirated, washed, and replaced with unbuffered DMEM. Unbuffered DMEM was also added to the four temperature-correction wells. The basal oxygen consumption rate (OCR) and extracellular acidification rate (ECAR) were measured using mito- and glyco-stress assay kits. In addition to the basal OCR, measurements were performed after the injection of the following four compounds affecting bioenergetics: oligomycin (1 μM; Sigma-Aldrich), carbonyl cyanide 4-trifluoromethoxy-phenylhydrazone (FCCP) (0.5 μM; Sigma-Aldrich), and a mixture of antimycin-A (1 μM; Sigma-Aldrich) and rotenone (0.5 μM). Furthermore, ECAR was measured following the successive addition of glucose (10 mM), oligomycin (1.0 μM) and 2-deoxy-D-glucose (2-DG, 50 mM; Sigma-Aldrich) (50 mM).

### Electron microscopy

The mitochondrial shape and localization were assessed by electron microscopy. Briefly, the cells were fixed by adding 0.2 ml of 2×fixative (1×concentration, 3% paraformaldehyde, and 2.5% glutaraldehyde in 0.1 M cacodylate buffer) at room temperature for 1 hour. The cells were centrifuged at 3200 RPM for 10 min at 30°C. The cells were washed with 200 μl of 0.1 M cacodylate buffer, stained with 200 μl of 2 mg/ml Evans Blue solution, and incubated for 20 min. The cell suspensions were transferred to 0.5 ml tubes and centrifuged at 3200 RPM for 10 min, and the buffer was pipetted. Then, 20 μl of 4% low melting agarose were added, and the tube was centrifuged at 3200 RPM for 10 min at 30°C and incubated at 4°C for 20 min to solidify. The cell pellet in solidified agarose was extracted, transferred through a 27 G needle to a 1.5 ml tube, washed 2–3 times with buffer, and observed under a transmission electron microscope.

### AP live staining

hBM-MSC treated with oocyte extract, and control cells were washed twice by Ca^2+^ and Mg^2+^-free phosphate-buffered saline (PBS) and colonies were mechanically dissociated. Colonies were added in ultra low-attachment dish in EB medium (Knock out DMEM, 20% Knockout Serum, 20% non-heat-inactivated fetal calf serum, 1% Non-Essential Amino Acid, 1 mM L-glutamine, and 0.1 mM b-mercaptoethanol) for 4 days [71]. Cells were then stained by AP live stain (Thermofischer Scientific, USA) according to the manufacturer’s instructions.

### Histology

Control and treated hBM-MSCs were fixed in 10% formaldehyde at room temperature for 24 hours. For histological and histochemical studies, paraffin blocks were prepared and 5-μm sections were used. Cells were stained by hematoxylin and eosin (HE) for histologic examination. To assess the ability of hBM-MSCs to differentiate into ectoderm, neurofibrils were stained by silver impregnation- luxol fast blue-periodic acid Schiff (LFB-PAS) stain. For mesodermal differentiation, safranin O was used to stain chondrocytes, while endodermal differentiation was assessed by PAS staining of hepatic cells.

### Protein extraction and trypsin digestion

Pooled oocyte cells of six biological replicates were lysed in 8 M Urea lysis buffer (500mM Tris pH 8.5) supplemented with cOmplete ULTRA, Mini, EDTA-free protease inhibitor cocktail (Roch, USA). The six samples were centrifuged at 10,000 RPM for 30 minutes at 4°C. Samples were subjected to quantification using bicinchoninic acid assay (BCA assay) (Pierce Thermo BCA, USA)). Thirty μg of each protein lysate samplewas reduced and alkylated with 200 mM Dithiothreitol (DTT) and 1 M Iodoacetamide (IAA), respectively. Denaturated protein samples were then diluted with 100 mM Tris pH 8.5 to reduce urea molarity to 2M prior to trypsinization. Proteins were digested at pH 8 with 1:30 (w/w) trypsin and incubated overnight at 37°C with shaking at 400 rpm (Biosan, USA). Digested peptides were desalted using C18 Monospin column (GL Sciences, Japan) [72].

### Nano-LC MS/MS analysis

Nano-LC MS/MS analysis was performed using TripleTOF 5600 + (AB Sciex, Canada) interfaced at the front end with Eksigent nanoLC 400 autosampler with Ekspert nanoLC 425 pump. Using CHROMXP C18CL 5um (10x0.5 mm) (Sciex, Germany) for peptide trapping on trap and elute mode. MS and MS/MS ranges were 400–1250 m/z and 170–1500 m/z, respectively. Gradient used in this experiment was 55-minute liner gradient 3–40% buffer B (80% ACN, 0.2% formic acid). The 40 most intense ions were sequentially selected under data dependent acquisition (DDA) mode with a charge state 2–5. For each cycle, MS and MS/MS spectra were acquired at resolution of 35.000 and 15.000, respectively. To ensure accuracy, a calibration was injected before the batch and within the batches scheduled to be every six hours to correct possible TOF deviation.

### Proteomics data analysis

Raw LC-MS/MS data in Wiff format were searched using the Paragon search algorithm ProteinPilot^™^ Software (Sciex, Germany). In this path, peptides were identified from their corresponding MS/MS spectra, then the Pro Group^™^ Algorithm (Sciex, Germany) was used for protein assembly. Database used for Homo sapiens (Swiss-prot and TrEMBL database containing 176507 proteins). The search space included all fully and semitryptic peptide candidates (up to 2 missed cleavages with at least 6 amino acids). Precursor mass and fragment mass were identified with an initial mass tolerance of 20 ppm and 10 ppm, respectively. Carbamidomethylation of cysteine (+57.02146 amu) was considered as a static modification and oxidation at methionine (+15.995), acetylation of protein N-terminal and K (+42.01 amu), and pyrrolidonefrom carbamidomethylated C (-17.03 amu) were considered as variable modifications. To ensure high-quality results, the false discovery rate (FDR) was maintained at 1% of the protein level.

The analysis is MIAPE compliant according to The Minimum Information About a Proteomics Experiment (MIAPE) reporting guidelines for proteomics of the HUPO Proteomics Standards Initiative (http://www.psidev.info/index.php?q=node/91), and our results adhere closely to the Paris guideline (http://www.mcponline.org/site/misc/ParisReportFinal.xhtml).

### Functional annotation enrichment analysis and pathogenic network inference

Functional enrichment analysis for the expressed proteins in the pooled oocytes was performed using Cytoscape (3.5.1) implemented with ClueGo (2.5.2). Enrichment analysis including molecular function and biological processes were obtained by a search of Gene Ontology annotation (GO) through ClueGo using the UniProtKB database (www.uniprot.org) and the EntrezPubMed database (www.ncbi.nih.gov) by searching unique gene symbol entries. Network analysis were generated by setting a medium network specificity using a GO term fusion.

### Statistical analysis

All data were statistically analyzed using SPSS 21.0 (SPSS, Chicago, IL, USA). Data were reported as mean ± SD. ANOVA with multiple comparisons post hoc tests was used for normally distributed quantitative variables while non-parametrical Kruscal-Wallis test and Mann-Whitney tests were used for non-normally distributed variables. Only at P < 0.05, results were considered significant.

## Supporting information

S1 Fig The human unfertilized stimulated oocytes. (A) Before the ultra-sonication, (B) After the ultra-sonication. Viewed via inverted microscope; scale bar (A) 50 μm, (B) 100 μm.
(TIF)

S2 Fig Flow cytomatric characterization of human bone marrow-derived mesenchymal storomal cells. Surface markers (A) CD105, (B) CD73 and (C) CD45 expressions.
(TIF)

S3 Fig Assessment of pluripotency, mesenchymal, and epithelial markers of human bone marrow-derived mesenchymal stromal cells control and treated by 10 ng/ul oocyte extract for 4 days. (TIF)

S1 Graphical Abstract (DOCX)
