# Supplementary figures and images for: Human-stimulated oocyte extract induces genetic and mitochondrial reprogramming of mesenchymal stromal cells
Source: PLoS One. 2020 May 26;15(5):e0232759. doi: 10.1371/journal.pone.0232759 (PMC7250418; doi:10.1371/journal.pone.0232759)

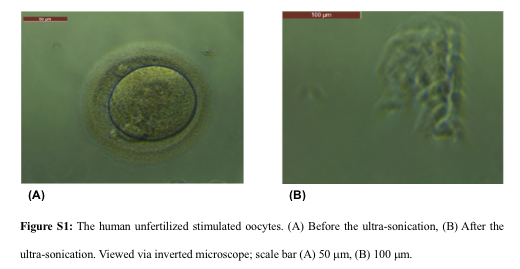

Supplement: S1 Fig — (A) Before the ultra-sonication, (B) After the ultra-sonication. Viewed via inverted microscope; scale bar (A) 50 μm, (B) 100 μm. (TIF) [file pone.0232759.s001.tif]

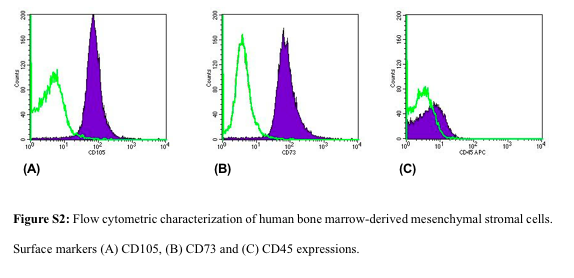

Supplement: S2 Fig — Surface markers (A) CD105, (B) CD73 and (C) CD45 expressions. (TIF) [file pone.0232759.s002.tif]

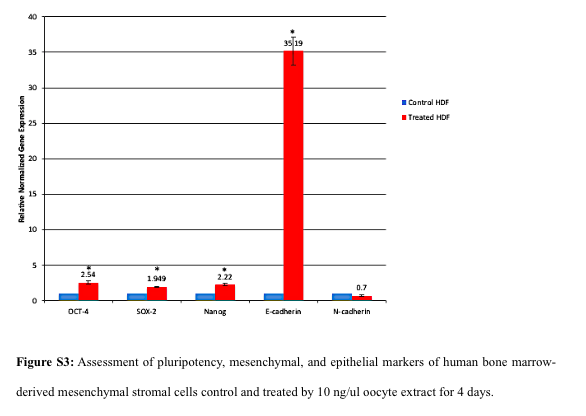

Supplement: S3 Fig — (TIF) [file pone.0232759.s003.tif]

Graphical Abstract

**
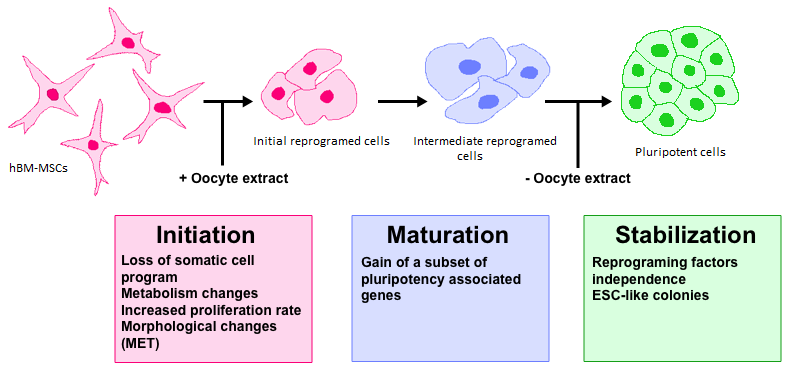
**

Supplement: S1 Graphical Abstract — (DOCX) [file pone.0232759.s004.docx]
